# Clinical trial protocol for P-NeLoP: a randomized controlled trial comparing the feasibility and outcomes of robot-assisted partial nephrectomy with low insufflation pressure using AirSeal versus standard insufflation pressure (UroCCR no. 85 study)

**DOI:** 10.1186/s13063-023-07533-4

**Published:** 2023-08-19

**Authors:** Gaelle Margue, Pierre Bigot, Alexandre Ingels, Morgan Roupret, Thibaut Waeckel, Jean-Alexandre Long, Géraldine Pignot, Karim Bensalah, Hervé Lang, Jonathan Olivier, Franck Bruyere, Matthieu Durand, Jean-Baptiste Beauval, Richard Mallet, Bastien Parier, Alexandre De La Taille, Jean-Christophe Bernhard

**Affiliations:** 1 Urology Department, Bordeaux University Hospital, Bordeaux, France; 2 Urology Department, Angers University Hospital, Angers, France; 3 Urology Department, Henri Mondor University Hospital, APHP, Paris, France; 4 Urology Department, Pitié-Saplétrière Hospital, APHP, Paris, France; 5 Urology Department, Caen University Hospital, Caen, France; 6 Urology Department, Grenoble Alpes University Hospital, Grenoble, France; 7 Urology Department, Paoli-Calmettes Institut, Marseille, France; 8 Urology Department, Rennes University Hospital, Rennes, France; 9 Urology Department, Strasbourg University Hospital, Strasbourg, France; 10 Urology Department, Lille University Hospital, Lille, France; 11 Urology Department, Tours University Hospital, Tours, France; 12 Urology Department, Nice University Hospital, Nice, France; 13 Urology Department, La Croix du Sud Hospital, Quint-Fonsegrives, France; 14 Urology Department, Polyclinique Francheville, Perigueux, France; 15 Urology Department, Kremlin Bicetre, APHP, Paris, France

**Keywords:** Insufflation pressure, Kidney cancer, Post-operative pain, Robot-assisted partial nephrectomy

## Abstract

Robot-assisted partial nephrectomy (RAPN) is the standard of care for small, localized kidney tumors. This surgery is conducted within a short hospital stay and can even be performed as outpatient surgery in selected patients. In order to allow early rehabilitation of patients, an optimal control of postoperative pain is necessary. High-pressure pneumoperitoneum during surgery seems to be the source of significant pain during the first hours postoperatively. Our study is a prospective, randomized, multicenter, controlled study which aims to compare post-operative pain at 24 h between patients undergoing RAPN at low insufflation pressure (7 mmHg) and those operated on at standard pressure (12 mmHg) using the AirSeal system.

This trial is registered in the US National Library of Medicine Trial Registry (NCT number: NCT05404685).

## Introduction and hypothesis

Kidney cancer is of increasing incidence in Europe and Worldwide. According to the Global Cancer Observatory, it is the 14th most common malignancy with 431,288 new cases in 2020 including 138,611 in Europe [1]. One explanation for this rising incidence is the increasing incidental diagnosis of small renal masses on imaging done for another cause [2, 3]. For these localized tumors (cT1-T2), conservative surgery (partial nephrectomy) is the standard of care [4], allowing good carcinological results while reducing morbidity. The minimally invasive approach with robotic assistance (robot-assisted partial nephrectomy (RAPN)) is now widely used in many hospitals. It combines a reduced perioperative morbidity and shorter hospital stays without altering the oncological results [5, 6]. This improvement in the procedure’s safety has allowed the development of Enhanced Recovery After Surgery protocols and ambulatory protocols [7–9]. Nevertheless, in general and in the context of outpatient care in particular, the control of postoperative pain is a crucial issue to allow convalescence and return to activity in the best possible conditions.

Laparoscopic procedures require the generation of a pneumoperitoneum by intra-abdominal insufflation of carbon dioxide (CO_2_) resulting in an impact on the cardiovascular and respiratory system (reduced venous flow and cardiac output, increased peripheral vascular resistance and blood pressure, increased pulmonary ventilatory resistance and hypercapnia) [10]. In order to limit these adverse effects, it is commonly recommended to limit the insufflation pressure to the minimum level necessary to allow sufficient exposure and working space. The commonly used insufflation pressure is 12–15 mmHg [10]. In addition to the side effects described above, this abdominal overpressure is also responsible for postoperative pain due to phrenic irritation and residual CO_2_ [11], which is, based on our expertise, mainly experienced during the first 48 h postoperatively and is difficult to control with the usual analgesics.

The AirSeal® system consists of an insufflator, a 3-channel tubing set, and a special trocar. It provides active, continuous, and self-regulated insufflation, suction and filtration of CO_2_. This allows the preservation of a stable pneumoperitoneum while ensuring continuous extraction and active filtration of electrocoagulation smoke [12].

This system has been shown to improve intra-operative ventilatory parameters during laparoscopic total cystectomy in a prospective randomized study [13]. Shahait et al. also reported a decrease in operative time and postoperative pain during robot-assisted total prostatectomy with the use of AirSeal at standard pressure in a non-randomized comparative study [14], while La Falce et al. demonstrated the feasibility of robot-assisted total prostatectomies at low insufflation pressure (8 mmHg) using the AirSeal® system [15]. Low-pressure insufflation is also being evaluated in other surgical areas. Therefore, Celarier et al. presented evidence of shorter hospital stay and decreased postoperative pain after laparoscopic colonic resection in a phase III randomized controlled trial evaluating the use of AirSeal at low pressure (7 mmHg) versus standard pressure (12 mmHg) [16]. Finally, specific data in the literature regarding the use of AirSeal® in RAPN is limited to the non-randomized prospective study of Annino et al. comparing standard insufflation with AirSeal® (at standard pressure of 12 mmHg) and reporting a benefit in terms of operating time, duration of renal ischemia, and rate of procedures without arterial clamping [17].

During the COVID-19 pandemic, the risk of contamination by surgical smoke, which may contain toxic gas but also viral pathogens, has been highlighted [18]. Their concentration within the abdominal cavity and the circulating insufflation gas result in a risk of aerosolization [19] and contamination of the operating theater and staff during laparoscopic procedures with or without robotic assistance [20]. This is especially significant in renal conservative surgery as SARS-COV-2 appears to have a capacity to bind to proximal convoluted tubules through its affinity for ACE2 receptors [20, 21]. This risk has led the EAU Robotic Urology Section (ERUS) committee to publish specific practice recommendations [22] advocating for the use of the lowest possible pneumoperitoneum pressure, active aspiration, and filtration of surgical smoke.

In this context, the P-NeLoP (partial nephrectomy in low pressure) trial aims to compare the effectiveness of AirSeal use at low pressure (7 mmHg), in terms of decreasing postoperative pain and therefore allowing fast re-autonomization of the patient, and its safety compared to its use at standard pressure (12 mmHg).

## Design

### Protocol overview

**Fig. 1:**
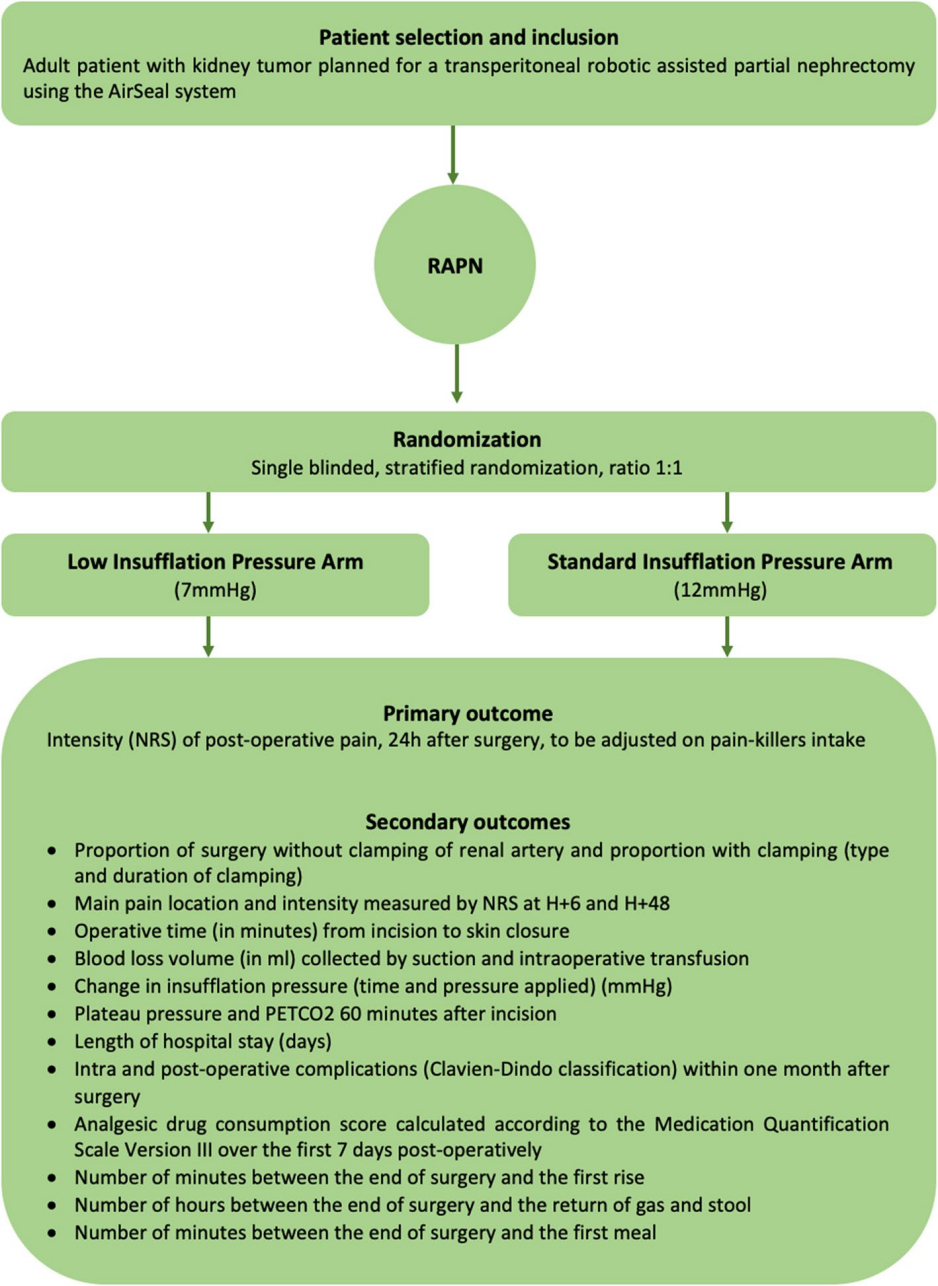
Study flowchart. RAPN, robot-assisted partial nephrectomy; NRS, numeric rating scale. Medication Quantification Scale Version III [23]

This is a two-arm, single blinded, prospective, multicenter randomized controlled trial that evaluates the impact of low insufflation pressure using AirSeal system (7 mmHg) during RAPN on post-operative pain, 24 h after surgery. We hypothesize that the use of AirSeal® at low pressure (7 mmHg) for RAPN would reduce post-operative pain, decrease the intake of analgesics, and thus facilitate patient re-autonomization. The study design is presented in Fig. 1.

### Study population and setting

This trial protocol is supported by the French research network on kidney cancer UroCCR (www.uroccr.fr; NCT03293563). Fifteen hospitals and clinics are involved in this multicentric study. The objective is to include 280 patients divided into two parallel groups: a control group undergoing surgery with a standard insufflation pressure (12 mmHg) and an experimental group undergoing surgery with a low insufflation pressure (7 mmHg), both using the AirSeal system. All adult patients managed for renal tumor and scheduled for transperitoneal RAPN are eligible for inclusion. The inclusion and exclusion criteria are summarized in Table 1.

**Table 1:** Inclusion and exclusion criteria

Inclusion criteria	Exclusion criteria
Male or female aged of 18 and over	Daily chronic painkillers intake for another indication than the kidney tumor and intended to be maintained at the time of surgery
Scheduled for transperitoneal RAPN with AirSeal system	Opioid substitution therapy
Affiliation to or beneficiary of the French social security	Person deprived of liberty
In capacity and willing to accurately report pain-killer intakes in the first postoperative 7 days	Person under trusteeship, curatorship, or legal guardianship
Free, informed, and written consent signed by the patient and the investigating physician (at the latest on the day of inclusion and before any examination required by the research)	Refusal of consent or participation in the UroCCR project and the P-NeLoP ancillary trial

*RAPN* Robot-assisted partial nephrectomy

This study was authorized and approved ethically by the Ouest I Ethics Committee on April 27, 2022 (ID-RCB: 2021-A03136-35), and is prospectively registered in the US National Library of Medicine Trial Registry (NCT number: NCT05404685).

### Assessment of baseline characteristics and inclusion

Patient characteristics, including medical history, blood work, imaging (including RENAL [24] and PADUA [25] nephrometry scores), and clinical data, will be collected by the investigating physician during diagnostic work-up and included in the web-based shared clinical and biological national database on kidney cancer UroCCR.

During the inclusion consultation, the patient will be fully informed by the investigating physician on the research protocol and given information notes on the P-NeLoP research trial and the UroCCR network. An informed consent form will be signed by each patient participating in the research protocol (Appendix).

### Randomization and blinding

Randomization will be performed just before surgery, with the patient being already under anesthesia. It will be conducted directly on the UroCCR database. After validation of the patient’s eligibility, the participant’s research number and the randomization result from the database, as well as the degree of insufflation, will be immediately communicated to the surgeon. The randomization list will be generated by the statistician at the Methodology and Data Management Center of the promoting hospital. The two groups will be balanced with a 1:1 ratio, and the randomization will be stratified according to the investigating centers. A detailed description of the analgesia habits of each center will be made at the beginning of the study.

A single blind will be applied. Patients will therefore not be informed of the group they have been allocated to.

### Procedure

All surgeries will be performed via transperitoneal robot-assisted approach with the use of an AirSeal device. The pressure level will be adjusted at the beginning of the surgery, after placement of the ports, according to the randomization result. In the event of technical difficulties experienced by the surgeon that may be induced by low pressure during a procedure performed at 7 mmHg, the pressure can be increased to the standard pressure of 12 mmHg. This information will then be collected as described in the secondary objectives.

### Intra- and post-operative follow-up

**Fig. 2:**
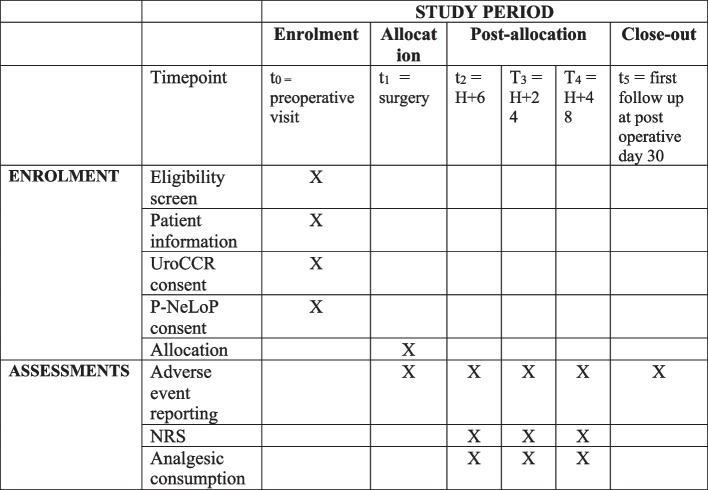
Timeline. NRS, numeric rating scale

The follow-up does not differ from the standard follow-up after RAPN. Four post-operative follow-up time points will be set to collect the data needed for evaluation (Fig. 2).

During surgery, complications, type of clamping, operative time, volume of blood loss, changes in insufflation pressure, and duration as well as the plateau pressure and PETCO_2_ 60 min after incision will be collected.

Post-operatively, we will record adverse events (according to Clavien-Dindo classification), presence of pain (according to the numeric rating scale (NRS)), and the use of analgesics (according to the Medication Quantification Scale Version III [23]), at H+6, H+24, and H+48. The return to eating, walking, and transit should also be noted. Finally, a first follow-up consultation is planned at day 30 (± 15 days) with collection of adverse events during the postoperative period.

## Statistics

### Calculation of the study size

We hypothesize that the average pain level measured at 24 h post-operatively by NRS will be 4.8 in the AirSeal® standard pressure group of 12 mmHg [11]. We also believe that the use of the AirSeal® device at a low pressure of 7 mmHg will decrease the mean pain by at least 0.8 points to 4.0 or less. With a common standard deviation of 2 [11], a power of 90%, an alpha risk of 5%, and a proportion of 5% missing or uninterpretable data, a total of 280 patients (140 patients per group) should be included (SAS® version 9.4, “Proc power” with a two-sided *t*-test on the difference in mean).

### Data analysis

The data will be analyzed by the biostatistician of the promoting hospital. The analyses will be performed with the SAS® software (version 9.4 or later). The main analysis will be performed on an intention-to-treat basis (any missing value of the primary endpoint will be replaced by the value corresponding to the failure of management, i.e., the maximum NRS value for all groups combined). Secondary objectives will be analyzed on available data.

A descriptive analysis will be performed overall and by group. The primary endpoint (NRS at H+24 postoperative) will be compared between the two randomization groups without adjustment and then after adjustment for the center and the level of analgesia prescribed (1, 2, or 3) using a linear regression model. Tests on the primary endpoint will be performed with an *α* risk of 5%.

## Trial status

The protocol number is ID-RCB: 2021-A03136-35, version no. 1.0 from 04/04/2022 promotor code: CHUBX 2020/62. The first inclusion was on October 4, 2022; the trial was scheduled to end around April 2024. The inclusion period is 18 months with 1 month of participation for the patient. The trial registration dataset can be found at https://clinicaltrials.gov/ct2/show/record/NCT05404685?term=NCT05404685&draw=2&rank=1.

## Conclusion

In recent years, we have witnessed an increase in cases of small renal tumors for which RAPN is now the standard of care. This surgery allows a shortening of hospital stay and is even performed on an outpatient basis for selected patients. Post-operative pain management is essential to the completion of these care paths and to the early rehabilitation of the patient. The AirSeal system allows the reduction of the insufflation pressure of the pneumoperitoneum during these surgeries which could lead to a significant decrease in postoperative pain. The P-NeLoP trial will evaluate the efficacy and safety of this low insufflation pressure device on a prospective basis.

## Data Availability

Data analysis will be performed by the principal investigator. This analysis will result in a written report which will be submitted to the sponsor, who will forward it to the Committee for the Protection of Persons and to the competent authority. Any written or oral communication of the results of the research must receive the prior agreement of the coordinating investigator and, where appropriate, of any committee set up for the research. The coordinating investigator undertakes to make available to the public all negative and inconclusive and positive research results. In accordance with the law no. 2002–303 of March 4, 2002, the participants will be informed, at their request, of the overall results of the research. The results of this study will be published in peer-reviewed journals and be presented at national and international conferences. The datasets analyzed during the current study and statistical code are available from the corresponding author on reasonable request, as is the full protocol.

## Appendix

### Patient consent form

#### UROCCR 85 – P-NELOP – CHUBX 2020/62

Research sponsor: CHU de Bordeaux

Coordinating investigator: Pr Jean-Christophe Bernhard

I, the undersigned .................................................................................................... (surname, first name), certify that I have read and understood the information note that was given to me.

I have had the opportunity to ask any questions I may have had to Professor/Dr .................................................................. (name, surname) who has explained to me the nature, objectives, potential risks and constraints of my participation in this research.

I am aware of the possibility of interrupting my participation in this research at any time without having to justify my decision and I will do my best to inform the investigator who is monitoring me in the research. This will of course not affect the quality of subsequent care.

I have been assured that the decisions that are necessary for my health will be made at any time, in accordance with the current state of medical knowledge.

I have been informed that this research has received a favorable opinion from the Committee for the Protection of Persons (CPP) and has been reported to the ANSM.

The Bordeaux University Hospital has signed a commitment to comply with the "Reference Methodology" (MR-001) in application of the provisions of article 54 paragraph 5 of the law of 6 January 1978 relating to information technology, files and freedoms, modified by

the law no. 2018-493 of 20 June 2018 on the protection of personal data.

The Sponsor of the research (CHU de Bordeaux, 12 rue Dubernat, 33404 Talence Cedex) has taken out insurance for liability insurance in case of damage with the company LLOYD'S INSURANCE COMPANY SA through the brokerage company BEAH SAS whose address is 16-18 rue de Londres, 75009 Paris (tel: 04 82 25 01 62).

I agree that the persons who collaborate in this research or who are mandated by the Promoter, as well as possibly the representative of the Health Authorities, may have access to the information in strict confidentiality.

I agree that the data recorded during this research, in pseudonymized (coded) form, may be subject to computerized processing under the responsibility of the Promoter. I am informed that, at the end of this research, these data may be used for other research purposes (new statistical analyses) and I have been informed of my right to object to this.

I have noted that, in accordance with the General Data Protection Regulation (RGPD) and the provisions of the law relating to data processing, files and freedoms, I have a right of access, rectification, deletion, limitation of processing, portability of data, opposition and withdrawal. These rights can be exercised with the investigator who is following me in this research and who knows my identity or via the UroCCR website www.uroccr.fr.

My consent does not relieve the investigator and the Sponsor of the research of their responsibilities to me. I retain all rights guaranteed by law.

The results of the study will be communicated directly via the UroCCR website www.uroccr.fr, in accordance with the law of 4 March 2002 on the rights of patients and the quality of the health system.

**Having had sufficient time to consider my decision, I freely and voluntarily agree to participate in P-NeLoP research.**

I freely and voluntarily agree that my clinical data may be stored and used for other research purposes (new statistical analyses). …. Yes … No

Done at ………... On: Done at ………….. On:

Name of investigator:

Signature of participant: Signature of investigator:
